# Visits to Private Asylums

**Published:** 1903-05-09

**Authors:** 


### VISITS TO PRIVATE ASYLUMS.

By Our Rambling Reporter.

### FISHERTON HOUSE, SALISBURY.

This, being licensed for 672 patients, is the largest private asylum or licensed house in Great Britain; and Dr. Finch, the present licensee, is the third generation of that name who has owned the institution. The original structure was known as Fisherton Manor House, and the existing buildings have been grouped around the old structure in more or less harmonious arrangement. It has now been an asylum for about sixty years. In position it is on the outskirts of the City of Salisbury, and is about ten or twelve minutes' walk from the station. The grounds are very extensive and amount to a total of 120 acres. The inmates here are not exclusively of the private class, as many of them are received under contract with the committees of public asylums or with Boards of Guardians, so that the institution partakes somewhat of the character of both a private asylum and a county one; but the large number of patients permits of their easy classification—a point of much importance. In fact it is on these grounds desirable that a licensed house should either be so small that the patients can be selected, and classification hardly needed, or it should be large enough to accommodate the various classes of the insane in separate divisions.

Speaking generally, it may be said that the day-rooms and dormitories are of smaller dimensions than usually met with in a county or public asylum containing nearly seven hundred beds; and this is a point in favour of Fisherton House, because, within certain limits, the smaller the ward the more homely will it be.

The County Asylum practice of placing all the epileptic cases under special observation at night is carried out here. There is an excellent recreation hall, and it is much used by the patients. A detached chapel, specially built in the grounds for their use, will seat about two hundred and fifty. The medical staff consists of Dr. Finch senior, Dr. Finch junior, and two assistant medical officers. This is ample for the number of patients usually in residence. The attendants and nurses would seem to be in the proportion of about one to seven patients which must also be looked on as amply sufficient.

